# Female genital mutilation among Iraqi Kurdish women: a cross-sectional study from Erbil city

**DOI:** 10.1186/1471-2458-13-809

**Published:** 2013-09-08

**Authors:** Berivan A Yasin, Namir G Al-Tawil, Nazar P Shabila, Tariq S Al-Hadithi

**Affiliations:** 1 General Directorate of Health, Erbil, Iraqi Kurdistan Region, Iraq; 2 Department of Community Medicine, College of Medicine, Hawler Medical University, Erbil, Iraqi Kurdistan Region, Iraq

## Abstract

**Background:**

Iraqi Kurdistan region is one of the areas where female genital mutilation is reportedly widely practiced but inadequately studied. The aim of this study was to determine (i) the prevalence of female genital mutilation among Muslim Kurdish women in Erbil city, (ii) the patterns and types of female genital mutilation, (iii) the factors associated with this practice and (iv) women’s knowledge and attitudes towards this practice.

**Methods:**

A cross-sectional study was conducted in the primary health care centers and the Maternity Teaching Hospital in Erbil city, involving 1987 women aged 15–49 years. Data were obtained about female genital mutilation status and knowledge and perception towards this practice. The participants were clinically examined to verify the self-reported female genital mutilation status.

**Results:**

The self-reported prevalence of female genital mutilation was 70.3%, while it was 58.6% according to clinical examination of the women’s genitalia. The most common type of female genital mutilation was type I (99.6%) and the most common age at which mutilation was performed was 4–7 years (60.2%). This practice was mostly performed by traditional birth attendants (72.5%). Only 6.4% of mutilated women reported having complications after mutilation, most commonly bleeding (3.6%). The practice was more reported among housewives (OR = 3.3), those women whose mothers were mutilated (OR = 15.1) or with unknown mutilation status (OR = 7.3) and those women whose fathers were illiterate (OR = 1.4) or could only read and write (OR = 1.6). The common reasons for practicing female genital mutilation were cultural tradition (46.7%) and dictate of religion (38.9%). Only 30% of the participants were aware about the health consequences of female genital mutilation. More than one third (36.6%) of the women support the practice and 34.5% have intention to mutilate their daughters.

**Conclusions:**

Prevalence of female genital mutilation among Muslim Kurdish women in Erbil city is very high; although, most cases are of type I. There is clear lack of knowledge about the health consequences of female genital mutilation and a relatively important segment of women support this practice. Custom or tradition and dictate of religion are the main reasons for this practice that need further in-depth exploration.

## Background

### Health consequences

Female genital mutilation (FGM) is associated with a series of health risks and consequences. It often causes pain and bleeding as immediate consequences of the procedure. Other immediate complications include difficulty in passing urine, infection including infection with human immunodeficiency virus, death that can be caused by hemorrhage or infections, unintended labia fusion and psychological consequences. Long term health risks include chronic pain, chronic infections, keloid formation, reproductive tract infections and sexually transmitted infections, increased risk of human immunodeficiency virus infection, poor quality of sexual life, birth complications and psychological consequences. Additional risks for complications particularly from type III include need for later surgery, urinary and menstrual problems, painful sexual intercourse and infertility [1-3].

### Violation of human rights

FGM is a clear violation of human rights of girls and women. It can be considered one of the main manifestations of gender inequality and discrimination related to historical suppression and subjugation of women, denying girls and women the full enjoyment of their rights and liberties [4].

### Epidemiology

FGM is a deeply rooted tradition in more than 28 African countries and a few populations in Asia and the Middle East [5]. It is estimated that 100–140 million women have experienced some form of the practice all over the world [6]. It is also estimated that around 3 million girls in sub-Saharan Africa, Egypt and Sudan, the majority of which below 15 years, are at risk of genital mutilation annually [5].

### Classification of FGM

The World Health Organization has classified the forms of FGM into four types. Type I, which is the mildest type, involves partial or total removal of the clitoris and/or the prepuce. Type I mutilation is subdivided into Type Ia, removal of the clitoral hood or prepuce only and Type Ib, removal of the clitoris with the prepuce. Type II involves partial or total removal of the clitoris and the labia minora, with or without excision of the labia majora. There are three major variations of type II mutilation; Type IIa involves removal of the labia minora only, Type IIb involves partial or total removal of the clitoris and the labia minora and Type IIc involves partial or total removal of the clitoris, the labia minora and the labia majora. Type III, the most severe type, involves narrowing of the vaginal orifice with creation of a covering seal by cutting and appositioning the labia minora and/or the labia majora, with or without excision of the clitoris (infibulation). Type III includes two subdivisions; Type IIIa involves removal and apposition of the labia minora and Type IIIb involves removal and apposition of the labia majora. Type IV involves all other harmful procedures to the female genitalia for non-medical purposes, for example: pricking, piercing, incising, scraping and cauterization [1].

In classifying FGM, the word ’clitoris’ is used to refer to the clitoral glans (i.e. the external part of the clitoris). It does not include the clitoral body or the crura, which are situated directly beneath the soft tissue and are not visible from outside. The clitoral prepuce is the fold of skin that surrounds and protects the clitoral glans [1].

### Legislations against FGM

Legislations prohibiting and criminalizing FGM have been introduced in several countries where FGM is practiced including several African countries [7]. Most industrialized countries, including the majority of Western Europe countries, where immigrant communities continue the practice have either employed already existing general criminal law provisions related to abuse or mutilation or introduced specific criminal law provisions prohibiting FGM [5,8].

### FGM in Iraqi Kurdistan region

FGM is widely practiced in Iraqi Kurdistan region, which is inhabited mostly by Muslim Kurds. According to activists and human rights organizations, the prevalence of FGM in Iraqi Kurdistan region is around 40% [9]. The roots of the practice in Kurdistan region are unclear. Although the practice is common in Iraqi and Iranian Kurdish areas [10], it is less common in other parts of Iraq and in Kurdish areas in neighboring Turkey. The prevalence of FGM is particularly high in the rural areas of Iraqi Kurdistan region. In some specific rural areas a prevalence of up to 70% has been reported. Traditionally, Kurdish society is agrarian; a significant part of the population lives outside cities, where the high prevalence of illiteracy and poverty and presence of conservative Islam appear to play a role in the high prevalence of FGM [9,11,12].

### Study objectives

There is little published evidence that has quantified the problem of FGM in Kurdistan region and described its patterns and associated factors. Moreover, there is lack of demographic and health survey data on Kurdish community and Iraq as whole. Therefore, this study was conducted to determine (i) the prevalence of FGM among Kurdish women in Erbil city, (ii) the patterns and types of FGM, (iii) the factors associated with this practice and (iv) women’s knowledge and attitudes towards this practice.

## Methods

### Setting and design

A cross-sectional study was conducted in Erbil city, the capital of Kurdistan region of Iraq, from November 2007 to March 2009. The study was carried out in the delivery rooms of the Maternity Teaching Hospital and the maternal care units of 14 primary health care centers in Erbil city.

### Study participants

A sample size of 1860 women was calculated based on having a ±3 precision around an estimated prevalence of FGM of 50% with a 95% confidence interval. The sample size was increased to 2000 to adjust for possible non-response. The study participants included married and unmarried Muslim Kurdish women of childbearing age (15–49 years). A convenience sample of 2000 women attending the primary health care centers and the delivery rooms of Erbil Maternity Teaching Hospital was selected. From each of the 14 primary health care centers in Erbil city, around 70 women were invited to participate in the study regardless of the catchment area of the health center. At the delivery rooms of Erbil Maternity Teaching Hospital, 1000 women were invited to participate in the study. Participants’ selection was made arbitrarily before knowing their FGM status.

### Data collection

Data were collected using a structured interviewer-administered questionnaire. The questionnaire addressed the main sociodemographic characteristics of the participants and the self-reported circumcision status of the participants and their mothers. The questionnaire also addressed the knowledge and attitude of the participants towards FGM. Here we used open questions to encourage participants to talk about FGM in terms of the reasons for practicing it, the known potential health consequences, whether they support this practice or not and whether they intend to mutilate their daughters or not. A female doctor verified the self-reported circumcision status of participants by clinical examination. Verbal informed consent was obtained from each participant prior to study enrollment. The study was approved by the Research Ethics Committee of Hawler Medical University.

### Data analysis

The statistical package for social sciences (SPSS, version 18) was used for statistical analysis. Proportions, means and tables were used for data summarization and presentation. Student’s t-test was used for comparing means. Univariate logistic regression was performed for assessing the association of sociodemographic factors with the practice of FGM. Multiple logistic regression for controlling for associated sociodemographic factors was performed for those variables that were statistically significant in the univariate logistic model. Degree of association was measured by odds ratio with 95% confidence interval. A p-value of ≤ 0.05 was considered as statistically significant.

## Results

Of the 2000 women, 1987 agreed to participate in the study with a response rate of 99.4%. The mean age ± SD of the participants was 27.6 ±6.9 years (range 15–49). Other sociodemographic characteristics of the participants are shown in Table 1.

**Table 1:** Sociodemographic characteristics of the participants

**Characteristic**	**No.**	**(%**)
**Age group (years)**		
15–19	193	(9.7)
20–29	1098	(54.9)
30–39	567	(28.4)
40–49	142	(7.1)
**Marital status**		
Married	1882	(94.7)
Single	95	(4.8)
Widowed	10	(0.5)
**Occupation**		
Housewife	1606	(80.8)
Employed (public or private sectors)	315	(15.9)
Student	66	(3.3)
**Place of birth**		
Urban	1492	(75.1)
Rural	495	(24.9)
**Mother education**		
Illiterate	1617	(81.4)
Read and write	89	(4.5)
Primary school	164	(8.3)
Intermediate school and higher education	117	(5.9)
**Father education**		
Illiterate	1002	(50.4)
Read and write	146	(7.3)
Primary school	431	(21.7)
Intermediate and higher school	408	(20.5)

Of the 1987 participants, 1397 (70.3%) reported to have undergone FGM. Clinical examination verified presence of FGM among 1164 (58.6%) participants. The mean age ± SD of clinically confirmed mutilated participants (27.8 ± 7.0 years) was significantly higher (P = 0.050) than that of non-mutilated (27.2 ± 6.9). Majority had type I mutilation (99.6%). Only five participants had type II mutilation; two were at the age of 15–19 years and three at the age of 20–29 years, all of them were born in rural areas, the mothers of four of them were illiterate and the father of one of them was illiterate. Only 983 (84.5%) mutilated participants remembered who performed mutilation for them. Of these, the majority of FGMs (72.5%) were performed by traditional birth attendants. Only 747 (64.2%) mutilated participants remembered the age at which they were mutilated. Of these, the majority (60.2%) were mutilated when they were between the ages of four and seven years. Around 6.4% of mutilated participants reported complications after mutilation; most commonly in the form of bleeding (3.6%). Details of the FGM characteristics of the clinically verified mutilated participants are shown in Table 2.

**Table 2:** Genital mutilation characteristics of clinically verified mutilated participants (n = 1164)

**Characteristic**	**No.**	**(%)**
**Type of FGM (n = 1164)**		
Type I*	1159	(99.6)
Type II**	5	(0.4)
**Person performed mutilation (n = 983)**		
Traditional birth attendant	713	(72.5)
Traditional circumciser	119	(12.1)
Relative	109	(11.1)
Neighbor	33	(3.4)
Health care providers	9	(0.9)
**Age at mutilation (years) (n = 747)**		
<4	124	(16.6)
4–7	450	(60.2)
8–11	158	(21.2)
12–15	14	(1.9)
≥16	1	(0.1)
**Complications after FGM (n = 1164)**		
**Short-term complications**		
Bleeding	42	(3.6)
Pain	11	(0.9)
**Long-term complications**		
Reduced libido	20	(1.7)
Psychological	2	(0.2)
**No complications**	1089	(93.6)

*FGM* female genital mutilation.

* Type I, partial or total removal of the clitoris and/or the prepuce (clitoridectomy).

* Type II, partial or total removal of the clitoris and the labia minora, with or without excision of the labia majora (excision).

The practice of FGM was significantly associated with the employment status of the women, FGM status of their mothers and education status of their fathers. The practice was more reported among housewives (adjusted OR = 3.3, 95% confidence interval (CI) 1.8-6.1), those women whose mothers were mutilated (adjusted OR = 15.1, 95% CI 10.6-21.6) or with unknown mutilation status (adjusted OR = 7.3, 95% CI 4.4-12.0) and those women whose fathers were illiterate (adjusted OR = 1.4, 95% CI 1.1-1.9) or could only read and write (adjusted OR = 1.6, 95% CI 1.02-2.5). No statistically significant association was found between the practice of FGM and the place of birth of the women or the education status of their mothers. Details of logistic regression for factors associated with the practice of FGM are shown in Table 3.

**Table 3:** Logistic regression for factors associated with the practice of FGM among participants

**Characteristic**	**FGM status of women**				**Crude**		Adjusted*	
	**Mutilated**		**Non-mutilated**		**Odds ratio (95% CI)**	**P value**	**Odds ratio (95% CI)**	**P value**
	**No.**	**(%)**	**No.**	**(%)**				
**Age group (years)**								
15–24	338	(43.7)	435	(56.3)	0.8 (0.6–1.0)	0.510		
25–34	339	(41.0)	488	(59.0)	0.9 (0.7–1.1)	0.279		
≥35	146	(37.7)	241	(62.3)				
**Occupation**								
Housewife	1024	(63.8)	582	(36.2)	4.7 (2.7–8.1)	< 0.001	3.3 (1.8–6.1)	< 0.001
Employed	122	(38.7)	193	(61.3)	1.7 (0.9–3.0)	0.081	1.8 (0.9–3.5)	0.075
Student	18	(27.3)	48	(72.7)				
**Place of birth**								
Rural	315	(63.6)	180	(36.4)	1.3 (1.1–1.6)	0.009	1.0 (0.8–1.2)	0.702
Urban	849	(56.9)	643	(43.1)				
**Mother FGM status**								
Mutilated	1067	(70.2)	453	(29.8)	18.5 (13.1–26.2)	< 0.001	15.1 (10.6–21.6)	< 0.001
Don’t know	57	(50.9)	55	(49.1)	8.2 (4.9–13.4)	< 0.001	7.3 (4.4–12.0)	< 0.001
Not mutilated	40	(11.3)	315	(88.7)				
**Mother education**								
Illiterate	1015	(62.8)	602	(37.2)	5.1 (3.3–7.9)	< 0.001	1.5 (0.9–2.6)	0.142
Read and write	45	(50.6)	44	(49.4)	3.1 (1.7–5.6)	< 0.001	1.4 (0.7–2.8)	0.344
Primary school	75	(45.7)	89	(54.3)	2.6 (1.5–4.3)	< 0.001	1.3 (0.7–2.5)	0.389
Intermediate school and higher education	29	(24.8)	88	(75.2)				
**Father education**								
Illiterate	654	(65.3)	348	(34.7)	2.7 (2.1–3.4)	< 0.001	1.4 (1.1–1.9)	0.022
Read and write	96	(65.8)	50	(34.2)	2.7 (1.8–4.1)	< 0.001	1.6 (1.02–2.5)	0.040
Primary school	246	(57.1)	185	(42.9)	1.9 (1.4–2.5)	< 0.001	1.3 (0.9–1.8)	0.110
Intermediate school and higher education	168	(41.2)	240	(58.8)				

*CI* confidence interval, *FGM* female genital mutilation.

* Only those variables that were significant in the univariate logistic model were considered for the multiple logistic regression model.

Only 29.5% of participants were aware that FGM could cause some complications. These participants identified reduced libido and bleeding as the most common complications; 23.9% and 3.6%, respectively. The participants cited social and cultural tradition (46.7%) and dictate of religion (38.8%) as the primary reasons for practicing FGM. More than one third (36.6%) of the women support the practice. Around 35% of participants have the intention to mutilate their daughters; 46.8% of mutilated participants and 17.1% of non-mutilated participants. Details of participants’ perception about FGM are shown in Table 4.

**Table 4:** Knowledge and perception of participants about FGM (n = 1987)

**Variable**	**No.**	**(%)**
**Impact of FGM on woman’s health**		
**No**	**1033**	**(52.0)**
**Don’t know**	**368**	**(18.5)**
**Yes**	**586**	**(29.5)**
Reduce libido	474	(23.9)
Bleeding	71	(3.6)
Pain	26	(1.3)
Infertility	4	(0.2)
Gloominess	3	(0.2)
Others (infection, difficult labor, etc.)	8	(0.4)
**Reasons for practicing FGM**		
Social and cultural tradition	928	(46.7)
Dictate of religion	770	(38.8)
Reduce libido	155	(7.8)
Cleanliness	14	(0.7)
Remove bad odor	7	(0.4)
More beautiful appearance	5	(0.3)
Don’t know	107	(5.4)
**Attitude towards FGM**		
Support FGM	728	(36.6)
Against FGM	1259	(63.4)
**Intention to mutilate daughters**	**686**	(**34.5**)
Mutilated women (n = 1164)	545	(46.8)
Non-mutilated women (n = 823)	141	(17.1)

*FGM* female genital mutilation.

## Discussion

FGM is a deeply rooted tradition that is still practiced in Erbil city. Cultural tradition and dictate of religion are the main provocative factors for continuation of the practice. According to UNICEF, Iraqi Kurdistan region and the Kurdish area of Iran have intermediate prevalence of FGM [4]. The high prevalence of FGM reported in this study was relatively lower than those reported by other studies from similar settings in Iraqi Kurdistan region; 63.0-72.8% in Erbil governorate [9,13], 73.0-77.9% in Sulaimania governorate [9,14] and 65.0-81.2% in Garmian and New Kirkuk region [9,15]. However, the reported rate of this study was in agreement with a study from Iranian Kurdish area in 2011 (55.7%) [10]. The above-mentioned studies from Iraqi Kurdistan region have primarily relied on self-declared FGM status in interviews, to determine the prevalence of FGM.

The relatively lower rate reported in this study might be attributed to verification of FGM by clinical examination rather than depending merely on interview results. A relatively high proportion of participants in this study (11.7%) reported that they have been mutilated but clinical examination revealed no mutilation. This does not necessarily mean that these women are not telling the truth. Under-reporting FGM is far more common than over-reporting [1,16]. In some societies women are expected to deny or under-report the type of FGM rather than over-reporting it. However, in other societies women might claim they are mutilated because of the high social pressure and the risk of social ostracism [17]. The disagreement between self-reporting and clinical examination can also be attributed either to having a very small cutting that could have been disappeared with anatomical growth of genital area or having made a small wound which was regarded as circumcision [18]. Disagreement between reporting FGM and examination findings has also been shown in a study from Egypt where 5% of women reported that they had been mutilated but were found not to have any evidence of FGM on examination. On the other hand, 1% of women had reported that they were not circumcised but some evidences of FGM were found on examination [19].

The reports of high prevalence of FGM in Iraqi Kurdistan region in 2007 resulted in launching the campaign of “Stop FGM in Kurdistan” by a number of civil society organizations and women’s rights groups. This awareness and advocacy campaign that was supported by a number of regional parliament members helped in adopting legal prohibition of FGM in Kurdistan region [20]. The substantial efforts that have been made in Kurdistan region to abandon this practice of FGM since 2007 resulted in passing the Family Violence Bill in June 2011. This bill includes several provisions criminalizing FGM in Kurdistan. The regional government also established the Women’s Affairs Supreme Council, a governmental agency directly linked to the Prime Minister’s office and responsible to combat all types of gender-based violence including FGM. These efforts have actively engaged civil society organizations and religious leaders to help in reducing the practice of FGM. These initiatives might have contributed to the increased awareness of the new generation about the health risks of FGM, which is evident from having a considerable proportion of the mutilated participants not having the intension to mutilate their daughters and the marginally statistically significant lower mean age of non-mutilated study participants than that of mutilated participants.

Broadly speaking, there is some evidence of increased awareness of the new generation about the health risks of FGM. In Egypt, the attitude about circumcision appears to be changing as the proportion of ever-married women who believe that circumcision should continue has dropped from 82 percent in 1995 to 63 percent in 2008 [21]. In Nigeria, similarly women younger than 36 years had more awareness about health risks of FGM than older women [22]. Moreover, women after marriage might change their attitudes towards FGM by following the customs of their husbands’ families that might not practice FGM in their tradition.

The main reasons for practicing FGM in this study were social and cultural tradition and dictate of religion. Moreover, a considerable number of study participants supported continuation of FGM practice, in particular the mutilated participants. This suggests that the main provocative factor for continuation of the practice is tradition and customs inherited in the family from mothers to daughters. In Egypt, which shares a relatively similar socio-cultural context to Iraq and Kurdistan region, the main reported reasons behind the practice are religious tradition (33.4%), cultural and social tradition (17.9%) and chastity (15.9%) [23]. Though no religious scripts prescribe FGM, it is often believed that the practice has religious support [23-25]. Religious leaders take varying positions with regard to FGM: some promote it, some consider it irrelevant to religion, and others contribute to its elimination [26]. For example, a reputed Islamic scholar in Iraqi Kurdistan region condemned the practice and completely denied any association between FGM and Islam [24]. As FGM is frequently linked to religious dictate, there should be an emphasis on the role played by religious leaders in fighting this phenomenon. In fact, the role of religious leaders in combating FGM in the region is extremely important due to the respect and influence they have in the local communities.

The most common type of FGM in this study was type I. In Egypt, type I and II are the most commonly reported types of FGM while in Africa type II accounts for up to 80% of all cases [23]. Studies that are based on clinical examination have documented large variations in the level of agreement between self-reported descriptions and clinically observed types of FGM [16,27-30]. Both under-reporting and less commonly over-reporting have been documented. The commonest discrepancy is that a large percentage of women declare that they have undergone Type I or II, even though clinical examination indicates Type III [30]. In addition, the reliability of clinical observation can be limited by natural anatomical variations and difficulty in estimating the amount of clitoral tissue under an infibulation [1].

In this study most of mutilations were carried out by traditional birth attendants, and a limited proportion by traditional circumcisers and health care providers. Unlike male traditional circumcisers for male circumcision that widely exist in the Iraqi Kurdish society, female traditional circumcisers rarely exist. Therefore, the traditional birth attendants, who usually have many duties besides attending deliveries of women at home, might consider genital mutilation one of their duties either as a religious obligation or as a custom to be practiced in their communities. According to different settings, different professions are involved in FGM. Unlike the Iraqi Kurdistan region, traditional circumcisers are responsible for performing most of the FGMs in the Iranian Kurdish area although the two areas share similar socio-cultural and religious contexts. Similarly, in Nigeria mutilation is mostly carried out by traditional circumcisers [10,31]. In other settings like Egypt, FGM is mostly performed by physicians [22,23]. According to the global strategy to stop health care providers from performing FGM, health care provider are responsible for performing more than 18% of all FGMs. In 2008, the World Health Assembly adopted a resolution on the elimination of FGM, in which all member states agreed to work towards the abandonment of FGM, including ensuring the procedure is not performed by health professionals [32]. However, health professionals continue to perform FGM in many settings [1]. In Erbil, the role of health care providers in performing FGM (0.9%) is much lower than other countries like Egypt (24-67%) [21,23] and Nigeria (11.9%) [33].

The age at which girls are mutilated varies among countries, ethnic groups and even regions within the same country [2,23]. Most participants in this study were mutilated when they were between the ages of four and seven years; a finding that agrees with a study from the Iranian Kurdish area where 54% of participants had the practice when they were below the age of seven years [10]. Another study from Sulaimania governorate reported an older age group (6–11 years) as the most common age of mutilation (60%) [14]. A study from Nigeria showed that 85% of women were mutilated when they were below one year [34] while in Egypt the mean age of the time of FGM is 10.1 years [23].

The extent of genital tissue cutting generally increases from Type I to III and the severity and risks are closely related to the anatomical extent of the cutting [1]. Therefore, the evidently low rate of complications reported in this study may be attributed to the high proportion of type I mutilation. The low complication rate could also be attributed to the fact that a relatively high proportion of women (35.8%) did not remember the event and reported no complications or that women do not link the complications to FGM due to their poor knowledge. Although reduced libido was reported as a complication of FGM, this could have other reasons than FGM such as marital status, depression and gynecologic problems. Unfortunately, this study did not investigate other possible causes of the reported reduced libido.

The practice of FGM was significantly associated with the mutilation status of the mother. Such a finding indicates that FGM is a custom that runs in the family as continuous practice from mothers to daughters. In a study in Indonesia from 2003, the majority of mothers of a sample of mutilated women reported themselves as being mutilated [35].

It is generally agreed that women’s education may contribute to a reduction of the practice [2]. Several other studies have reported a negative association between FGM and the education level of mothers [4,10,19,23]. However, this study did not show statistically significant association between FGM and the education status of the mother. Such findings might suggest that education alone is not sufficient to lead to the abandonment of FGM and might signal the superiority of traditions, cultural inherits and religious dictate over education in the Kurdish society. Interestingly, this study showed a statistically significant association between FGM and the education status of the father, which agrees with a study from Egypt [23]. This may reflect the decision making process on FGM in the family and the society and the potential power of the father in making such decision. The role of father in making such decisions has not been studied in the Kurdish society so far. This issue needs further exploration and the potentially effective role of father should be considered in prevention programs. Research from other settings like Gambia has shown that the decision making for undergoing FGM is in large part made by mothers. However, there are instances where it is a joint decision by both mother and father with the latter only informed to obtain his agreement. Other decision-makers are female members in the family, particularly grandmothers [36].

### Study limitations

This study was not able to identify and report the exact sub-types of FGM among the participants. It also does not cover the rural areas where a higher prevalence of FGM is expected. Future research should focus on the prevalence of the complications associated with FGM and the exploration of the roots of FGM in the Kurdish community in an in-depth manner.

## Conclusions

Prevalence of FGM in Erbil city is very high; however, most cases are of type I. The practice is significantly associated with the employment status of the women, FGM status of their mothers and education status of their fathers. There is clear lack of knowledge about the health consequences of FGM. There is still a relatively important segment of women who support FGM and intend to mutilate their daughters. This signals the need for extensive education programs that could be carried out by health and education authorities in addition to religious leaders. Custom or tradition and dictate of religion are the main reasons for FGM that need further in-depth exploration.

## Abbreviations

CI: Confidence interval; FGM: Female genital mutilation.

## Competing interests

The authors declare that they have no competing interests.

## Authors’ contributions

BAY and NGAT conceptualized the study. BAY, NGAT and TSAH participated in designing the study. BAY, NGAT and NPS collected the data and carried out data analysis. BAY and NPS drafted and finalized the manuscript. TSAH and NGAT extensively reviewed and edited the manuscript. All authors contributed to interpreting study results and writing the manuscript. All authors read and approved the final manuscript.

## Pre-publication history

The pre-publication history for this paper can be accessed here:

http://www.biomedcentral.com/1471-2458/13/809/prepub
